# What do we know about blood-testis barrier? current understanding of its structure and physiology

**DOI:** 10.3389/fcell.2023.1114769

**Published:** 2023-06-15

**Authors:** J. P. Luaces, N. Toro-Urrego, M. Otero-Losada, F. Capani

**Affiliations:** ^1^ Centro de Altos Estudios en Ciencias Humanas y de la Salud, Universidad Abierta Interamericana, Consejo Nacional de Investigaciones Científicas y Técnicas, CAECIHS.UAI-CONICET, Buenos Aires, Argentina; ^2^ Instituto de Ciencias Biomédicas, Facultad de Ciencias de la Salud, Universidad Autónoma de Chile, Santiago, Chile

**Keywords:** blood-testis barrier (BTB), spermatogenesis, adherens junction, tight junction, gap junction, high-resolution microscopy

## Abstract

Blood-testis barrier (BTB) creates a particular compartment in the seminiferous epithelium. Contacting Sertoli cell-Sertoli cell plasma membranes possess specialized junction proteins which present a complex dynamic of formation and dismantling. Thus, these specialized structures facilitate germ cell movement across the BTB. Junctions are constantly rearranged during spermatogenesis while the BTB preserves its barrier function. Imaging methods are essential to studying the dynamic of this sophisticated structure in order to understand its functional morphology. Isolated Sertoli cell cultures cannot represent the multiple interactions of the seminiferous epithelium and *in situ* studies became a fundamental approach to analyze BTB dynamics. In this review, we discuss the contributions of high-resolution microscopy studies to enlarge the body of morphofunctional data to understand the biology of the BTB as a dynamic structure. The first morphological evidence of the BTB was based on a fine structure of the junctions, which was resolved with Transmission Electron Microscopy. The use of conventional Fluorescent Light Microscopy to examine labelled molecules emerged as a fundamental technique for elucidating the precise protein localization at the BTB. Then laser-scanning confocal microscopy allowed the study of three-dimensional structures and complexes at the seminiferous epithelium. Several junction proteins, like the transmembrane, scaffold and signaling proteins, were identified in the testis using traditional animal models. BTB morphology was analyzed in different physiological conditions as the spermatocyte movement during meiosis, testis development, and seasonal spermatogenesis, but also structural elements, proteins, and BTB permeability were studied. Under pathological, pharmacological, or pollutant/toxic conditions, there are significant studies that provide high-resolution images which help to understand the dynamic of the BTB. Notwithstanding the advances, further research using new technologies is required to gain information on the BTB. Super-resolution light microscopy is needed to provide new research with high-quality images of targeted molecules at a nanometer-scale resolution. Finally, we highlight research areas that warrant future studies, pinpointing new microscopy approaches and helping to improve our ability to understand this barrier complexity.

## Introduction

Since the first contributions to the field of spermatogenesis, microscopy-based studies were fundamental to understanding cell composition and associations in the seminiferous epithelium (França et al., 2016). The spermatogenic parenchyma is an intricate environment due to its tubular organization with specific cell associations along the seminiferous tubules. Histological cross-sections of mammals’ testis show a variety of germ cell maturation stages, from tubules, plenty of well-developed spermatids (almost spermatozoa), to tubules with immature spermatids in contact with tubule lumen (Nishimura and L’Hernault, 2017).

Blood-testis barrier (BTB) is a complex cell structure present in the seminiferous epithelium. It creates two tissular compartments: the basal one, with spermatogonia and early spermatocytes; and the adluminal one, with spermatocytes and post-meiotic germ cells (Stanton, 2016). BTB was first described due to its physiological function by analyzing the rate of passage of different substances from plasma into the rete testis (Setchell et al., 1969). When specific tracers are perfused through the testicular artery, the adluminal compartment remains spared (Pelletier, 2011a). The nurturing Sertoli cells build particular interconnections, creating an isolated adluminal compartment. During the active phase of spermatogenesis, germ cells differentiate and drive across the BTB. This process is unequivocally dynamic with cells projecting to contact others, and adhesion molecules being rearranged precisely to admit the passage of germ cells without affecting the barrier permeability (Mruk and Cheng, 2015). This complex event challenges studying BTB cellular and molecular biology, and actually, no *in vitro* models can represent the situation *in situ* where multiple cell types interact synchronously (Mruk and Cheng, 2012).

In the mammalian testis, specific cell associations occur along the seminiferous tubule. Depending on maturation, germ and Sertoli cells are exposed to different microenvironments and physiological conditions. Under these stimuli, BTB junctions are rearranged through a dynamic and precise spatio-temporal process (Xiao et al., 2011).

The first morphological shreds of evidence of a cellular barrier in the testis were based on conventional Transmission Electron Microscopy (TEM) (Dym and Fawcett, 1970). Further research was largely accomplished using animal models exploring the biology of the BTB *in situ*. Still, the current knowledge of junctions’ dynamics and regulatory factors like cytokines, proteases, and androgens mainly comes from *in-vitro* models using Sertoli cells. After the refinement of immunolocalization techniques and the setup of Sertoli cell cultures, a substantial number of studies on the Sertoli cell barrier’s functional morphology were based on *in vitro* models (Mruk and Cheng, 2011). Cytological preparations obtained from cell cultures allow for the precise, thorough study of subcellular processes and the localization of specific proteins. The study of the cellular and molecular dynamic of the BTB *in situ* demands working with animal models and optimizing sample preparation methods, yet allowing the investigation of the complexes and assemblies at the BTB in their tissular environment.

Here, we review the contributions of high-resolution microscopy (HRM) to the advancement in the study of BTB cellular and molecular morphology, based on animal model studies that analyze the *in situ* barrier biology. We overview several studies on BTB functional morphology under different physiological and pathological situations. Finally, we discuss future directions in the study of the BTB with new technologies of high-resolution microscopy.

Considering the complexity of the seminiferous epithelium, to focus on the barrier function of Sertoli cells in the testis, only the studies regarding the junctional complex at the Sertoli cell–Sertoli cell interphase of the BTB *in situ* were considered.

## Background: Mammalian spermatogenesis and BTB

Spermatogonia undergo spermatogenesis evolving to mature spermatids through a complex process, and finally spermatozoa are produced (Nishimura and L’Hernault, 2017). The study of the cellular biology of the seminiferous epithelium is challenging due to the multiple cell associations taking place in the active phase of spermatogenesis (Leblond and Clermont, 1952). Through the different stages of the seminiferous epithelium during active spermatogenesis, the variety of cell types make up a drastically ever-changing spermatogenic parenchyma. Concurrently, the BTB undergoes changes shaping a dynamic and intricate physical barrier.

1. Structural features of the BTB

**FIGURE 1:**
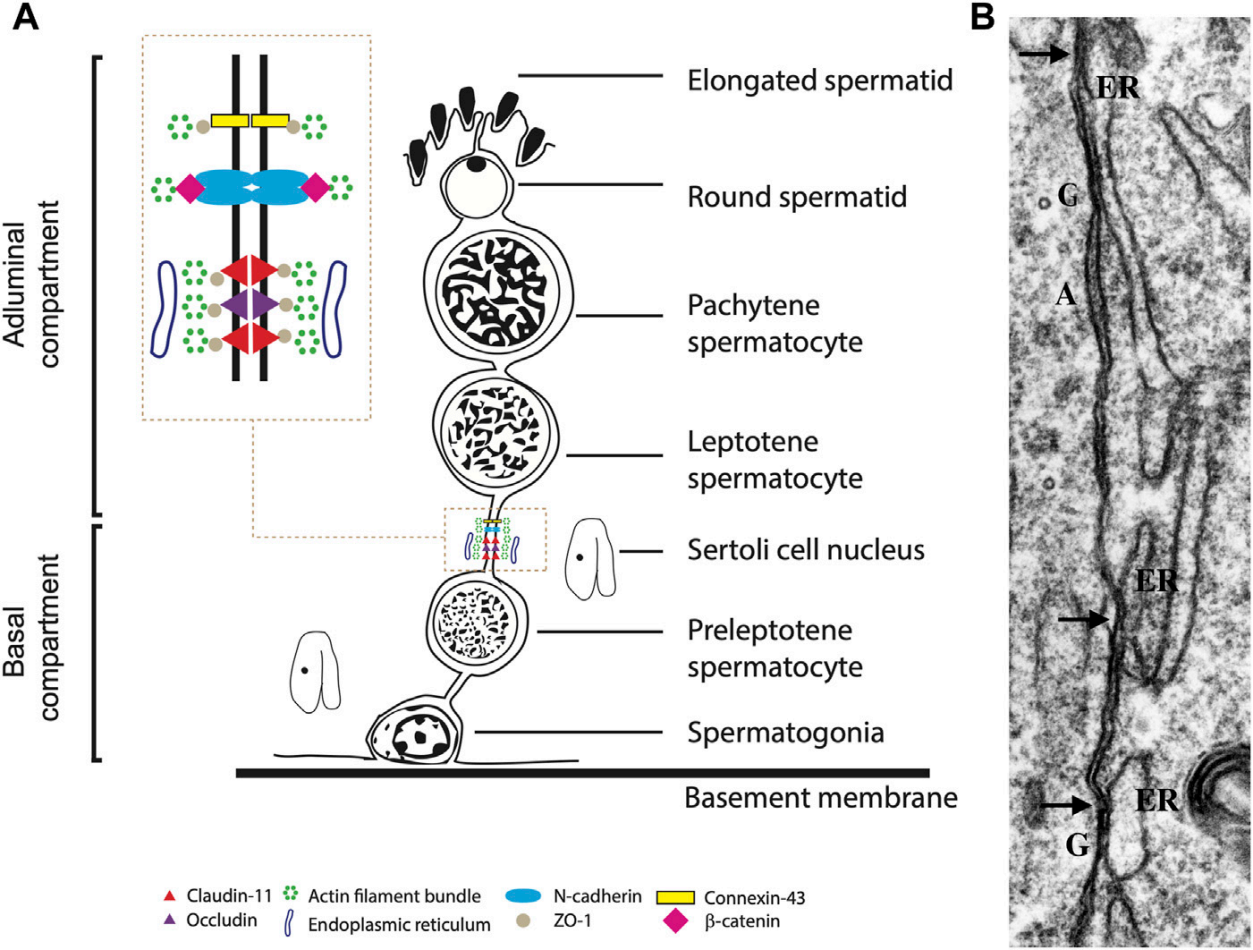
**(A)**. Diagram illustrating classical protein complexes at the typical junction of the blood-testis barrier in the seminiferous epithelium. **(B)**. Transmission electron microscopy of Sertoli cell - Sertoli cell junction elements during the reactivation of testis during the annual reproductive cycle in the mink; highlighting the contacts of the ER (arrows) with elements of the cell junctional complexes. A: adhering junction; G: gap; arrow: Sertoli-Sertoli tight junctions. X 34,000. Figure partially reproduced with permission from Pelletier (2011a).

The BTB is mainly made up of widespread tight junctions (TJs) between somatic Sertoli cells forming paracellular seals close to the basement membrane (Dym and Cavicchia, 1977; Pelletier and Byers, 1992; Pelletier, 2011a) (Figure 1). With scanning electron microscopy, TJs look like strands in freeze-fracture preparations, being long and short strands, running parallel, or crosslinking among them (Pelletier, 1986; Ribeiro and David-Ferreira, 1996). Between TJ strands, adherens and gap junctions are present (Pelletier et al., 2011, Figure 1B). In association with Sertoli cell TJ, ectoplasmic specializations (ES) occur as zones at the periphery of the cell, with actin filament bundles hexagonally packed and endoplasmic reticulum cisternae parallelly disposed to the Sertoli cell membrane. Figure 1A shows BTB junctions’ major components and specific proteins.

Although desmosome elements have yet to be identified using antibodies (Domke et al., 2014), various authors have defined “desmosome-like contacts” at the ultrastructural level (Russel, 1977; Russell and Peterson, 1985; Vogl et al., 1993). Pelletier and Byers (1992) redefined these unions as “intermediate type of adherin junctions”. Domke et al. (2014) could not target desmosome-specific proteins at the seminiferous epithelium but found them in other epithelia of the testicular excurrent ducts.

2. Electron microscopy studies

Before immunolocalization, electron microscopy allowed the fine study of the BTB. There are both transmission and scanning microscopy studies, the latter of which have made studying the lateral surface of the Sertoli cell possible.

Correlative TEM and freeze-fracture microscopy studies were used to understand the nature of TJ at the BTB, and to distinguish whether a TJ was a focal contact or a seal with a barrier function (Pelletier, 2011a). The surface of the lateral cleaved plasma membrane was visualized in very elegant studies carried out in different species (Nagano and Suzuki, 1976; Russell and Peterson, 1985; Pelletier, 1986; Cavicchia and Sacerdote, 1988; Kan and Lin, 1995). With freeze-fracture microscopy, TJ fibrils showed to be continuous strands in the protoplasmic face (P-face) and complementary grooves in the extracellular face (E-face) (Morita et al., 1999). In the mink, correlative MET and freeze-fracture studies showed that BTB formation during testis development was characterized by progressive basal TJ assembly. Strands of particles were first observed isolated and short in the prepuberal testis, and after testis maturation strands were made of multiple continuous rows of particles disposed roughly parallel to each other and to the basal lamina to make a competent BTB (Pelletier, 1986), coincident with similar studies in rats (Cavicchia and Sacerdote, 1991). The fracture-exposed junction particles were associated with either the P or the E fracture face in prepuberal testes, while particles were preferably found in the E-face in the adult (Pelletier, 1986). Discontinuous rows were associated with an increase in BTB permeability to substances from the interstitial space to the tubular lumen (Morales et al., 2007).

Immunoelectron microscopy carried out in fracture replicas revealed oligodendrocyte-specific protein (OSP)/claudin-11 distributed on the fully formed TJ strands of Sertoli cells (Morita et al., 1999) and over the seminiferous epithelium in the mouse testis (Hellani et al., 2000).

3. Localization of single molecules and adhesion protein complexes in the BTB

In this section, reports showing high-resolution images of the immunolocalization of structural proteins of the BTB and others that are part of the signaling that regulates the junction complexes and the permeability of the barrier are mentioned.

Targeting of single proteins in the testis by immunofluorescence (IF) and immunohistochemistry became a key tool for understanding the architecture of body tissues. After the 90s, massive use of this technique was reported in several publications (Hogarth and Griswold, 2013). Then, laser-scanning confocal microscopy enhanced this technique by allowing the study of three-dimensional structures and complexes at the seminiferous epithelium (França et al., 2016).

The TJs in the Sertoli cell membranes embody the structural basis of the BTB. BTB’s TJs are composed of two main transmembrane proteins, claudin-11 and occludin. These integral proteins comprise the skeleton of the TJs and are arranged as particles along junctional strands, making up for only a small portion of the intramembranous particles at the junctional fibrils (Morita et al., 1999). Adherens and gap junctions at the BTB were also well analyzed with different models (Mulholland et al., 2001; Johnson and Boekelheide, 2002b; Domke et al., 2014).

The TJ structure comprises a transmembrane region with identical transmembrane proteins attached to both sides (Mruk and Cheng, 2010). Besides, scaffolding proteins at the cytoplasmic side of the tight junction link to the actin cytoskeleton and are involved in signaling pathways, controlling junction structure and function (Lui et al., 2003, Figure 1A).

Claudins are the major TJs’ integral transmembrane proteins (Morita et al., 1999; Jiang et al., 2014). The distribution of claudin-3 and -11 and occludin was studied along different stages of the seminiferous epithelium in a mouse model for conditional androgen insensitivity (Meng et al., 2005). In the Iberian mole, clau-11 expression pattern was analyzed across the seminiferous cycle stages showing a cycle-dependent distribution with the strongest signal observed parallel to the basal membrane in stages VII-VIII (Dadhich et al., 2013).

Zonula occludens-1 (ZO-1) is an adaptor protein that links transmembrane proteins to the actin cytoskeleton. The position of the scaffold cytoplasmic protein ZO-1 was well characterized in several studies (Chihara et al., 2010; Mok et al., 2011b; Schimenti et al., 2013). It was identified at the apical membrane of the Sertoli cells in contact with mature spermatids in the BTB (Byers et al., 1991).

Focal adhesion kinase (FAK) is a major regulator of the BTB as a non-receptor protein kinase (Lie et al., 2012; Li et al., 2013). At the BTB, FAK links to the occludin-ZO-1 complex and regulates actin dynamics by converting from bundled to unbundled filaments at the ES. Gene knockdown of FAK produces the alteration of occludin phosphorylation status and occludin-ZO-1 association, destabilizing the Sertoli cell TJ-permeability barrier (Siu et al., 2009a). Stage-specific localization of FAK and co-localization with occludin was found at the basal membrane of Sertoli cells (Siu et al., 2009b).

Occludin is an integral membrane protein constitutive of tight junctions. In adult mice and rat testis, occludin is distributed in a linear pattern in the basal regions of the Sertoli cells’ TJ strands. Also, its distribution overlapped with ZO-1 at the basal third of the Sertoli cells (Morita et al., 1999).

Key structural and regulator proteins of the BTB (ZO-1, β-catenin, claudin-11, and Par-6 family cell polarity regulator beta, PARD6B) showed varying localization according to germ cells’ differentiation stage in a mutant infertile Kit^w^/Kit^wv^ mice model which received spermatogonial stem cell transplant (Li X. Y et al., 2018).

Beta-catenin is a plaque protein associated with cadherin, found in the basal Sertoli cell cytoplasm (Dadhich et al., 2013; Domke et al., 2014; Li X et al., 2018; Merico et al., 2019).

At the cadherin-based junctions, p120 is attached to cadherin in the cytoplasmic domain. In the rat testis, p120 was observed at the basal junctions by immunolocalization, but not associated with ectoplasmic specialization (Johnson and Boekelheide, 2002a). In the adult testis, p120 distribution was overlapped with N-cadherin at the base of the seminiferous epithelium (Johnson and Boekelheide, 2002b).

Plectin, a plakin protein. Is found at the basal interface in Sertoli cell-cell junctions. Plectin co-locates with vimentin at the end of the intermediate filament and is distributed as focal patches (Guttman et al., 1999).

N-cadherins are transmembrane adhesion proteins (Nollet et al., 2000) found in the TJ between the Sertoli cells. N-cadherin showed a stage-specific distribution in the rat testis located basally in the Sertoli cell-cell junction and juxtaposed though not colocalized with basal ectoplasmic specialization (Mulholland et al., 2001; Johnson and Boekelheide, 2002b).

Cortactin is an F-actin filament-binding protein linked to intercellular junctions. At the junction, cortactin is associated with the junction proteins N-cadherin and E-cadherin, and accessory proteins like ZO-1, p120-catenin, and β-catenin (Vitale et al., 2009). In the adult mice, P-cortactin was identified at the base of the Sertoli cell cortical cytoplasm near plasma membrane segments, showing a stage-dependent distribution. Depending on the state, cortactin was found above the spermatogonia (stage V), surrounding spermatogonia and preleptotene spermatocytes (stage VII), or close to the basal membrane (stage VIII and X). Cortactin and Cx43 were found as linked molecules and their colocalization in the basal third of the seminiferous epithelium was stage-dependent (Vitale et al., 2009).

Another member of the Claudins family, claudin-5, has been reported to contribute to BTB function in mice. It was largely expressed along the different stages of the seminiferous epithelium with strong staining at stage VIII, nearby preleptotene, and leptotene spermatocytes (Morrow et al., 2009).

Other constituents of the BTB, like the transmembrane junctional adhesion molecule 1 (JAM-1) and the Coxsackie and adenovirus receptor (CAR), were studied *in situ* (Mruk and Cheng, 2004; Wang et al., 2007; Lie et al., 2012).

Filamentous actin (F-Actin), another BTB component of the basal ES, was localized in the seminiferous epithelium and actin dysregulation was observed in a non-collagenous (NC) 1 domain peptide overexpression model. The structure of actin and microtubule cytoskeleton, and the localization of regulatory proteins were analyzed, and alterations were also associated with NC-1 peptide overexpression (Liu et al., 2020). Calmodulin-regulated spectrin-associated protein (CAMSAP), a microtubule tracking protein, showed a prominent stain at the BTB near the base through the different stages of the seminiferous epithelium (Mao et al., 2019).

Other regulators have been also identified. Fyn immunoreactivity was detected in Sertoli cells along the different stages of the seminiferous epithelium, concentrated at the basal sites of the epithelium, where ESs are found (Maekawa et al., 2002).

Cell surface proteins like the single transmembrane coxsackievirus and adenovirus receptor (CAR) are present in most epithelial TJs. CAR was found in the basal compartment in all rat testis stages. During fully active spermatogenesis, CAR and ZO-1 co-localized near the base of the seminiferous epithelium, showing that both are constituents of the BTB (Wang et al., 2007).

Connexin (Cx) 43 is the principal protein of the gap junction (GJ) of the testis. GJs are typical BTB constituents found at the contact membrane between Sertoli cells (Risley et al., 1992; Pointis and Segretain, 2005). At the base of the epithelium, Cx43 colocalized with occludin (Cyr et al., 1999; Carette et al., 2010).

Two studies showed stage-specific localization proteins involved in actin and microtubule cytoskeleton regulation (Su et al., 2012). Scribble and ZO-1 were co-localized in the BTB (Su et al., 2012). Dishevelled-3 was targeted together with F-actin and α-tubulin, indicating a close association of regulatory and cytoskeletal proteins (Li et al., 2019). It has been shown that the polarity complex Scribble/Lgl (lethal giant larvae)/Dig (discs large) affects the ES dynamic, regulating the actin filament network (Su et al., 2012).

Intermediate filaments are one of the three major elements of the cell cytoskeleton and are associated with cell junctions at Sertoli-Sertoli cell junctions. The localization of vimentin and other non-typical intermediate filaments as glial fibrillary acid protein (GFAP) and neurofilaments (NF) were targeted in the Sertoli cell cytoplasm (Aumüller et al., 1992; Davidoff et al., 1999; Budipitojo et al., 2018). Vimentin filaments distribution changed with the stage of spermatogenesis, with the shortest filaments found during early spermiation stages (Show et al., 2003). Assembled NF filaments were localized as the three filament forms (NF-L, NF-M, and NF-H) in the human testis (Davidoff et al., 1999).

4. Current limitations of *in vitro* Sertoli cells’ models

In preparations using an appropriate substrate, Sertoli cells maintain morphological characteristics and establish TJ complexes similar to those present *in situ* (Mruk and Cheng, 2011). Transepithelial resistance of *in vitro* systems is lower than expected for the BTB, attributing this difference to the absence of germ and peritubular myoid cells (Mruk and Cheng, 2015). The highly complex tissular environment in the testis may partially explain why, so far, no *in vitro* co-culture model has mimicked germ cell migration across the BTB. While a variety of factors regulate Sertoli cell junctions, evidence continues to be collected from experiments using Sertoli cells cultured alone (Mruk and Cheng, 2012). Considering that no *in vitro* model actually reflects the role of germ cells in regulating BTB, all data from Sertoli cell cultures should be analyzed with reservations concerning that: BTB is a highly dynamic structure *in situ* and the limits of extrapolation of *in vitro* data to the whole animal (Saino and Satoh 2009). Since the possibility of tracking live events using cell culture and the accuracy of immunolocalization performed on cytological preparations, new models *in vitro*, including co-culture of Sertoli and germ cells, are needed that more closely relate them with *in-vivo* BTB models.

5. BTB junction assembly during testis development

**FIGURE 2:**
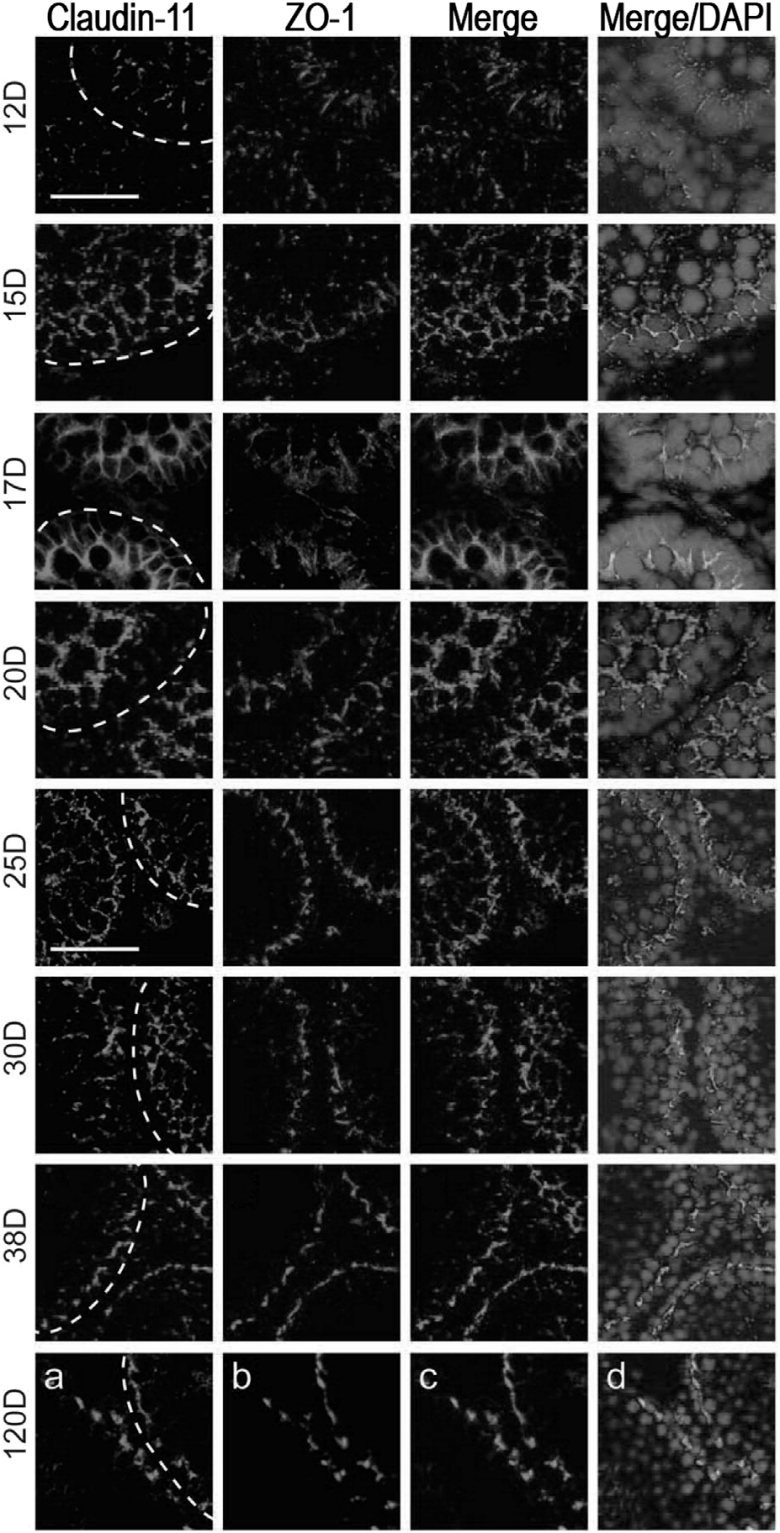
Blood-testis barrier morphology during testis development in rats. Claudin-11 (red) and ZO-1 (green) co-localization in testes sections obtained after 12-, 15-, 17-, 20-, 25-, 30-, 38- and 120 days *postpartum* (dpp). Proteins co-localized at the BTB from day 25, showing junction assembly. Bar 25 μm: micrographs of 12–20 dpp; Bar 50 μm: micrographs of 25–120 dpp. Figure reproduced partially with permission from Mok et al. (2011) and modified.

Soon after mice birth, gonocytes attach to the basal membrane and become spermatogonia, occupying their definitive localization, the basal compartment (Mirza et al., 2007). Testicular cords during embryonic development present no complete spermatogenesis and no functional BTB. Sertoli cell TJs appear during fetal life, then a few discontinuous junctional fibrils can be observed in juveniles while continuous fibrils develop in puberty with the establishment of the BTB (Morales et al., 2007). Fully functional BTB settles in puberty with the assembly of continuous fibrils at the junctions, giving rise to TJ zonules sealing the space between Sertoli cells (Pelletier and Friend, 1983; Pelletier, 2011a). Sertoli junctions in the adult testis adapt to the cyclic passage of meiotic cells while maintaining TJ function. In rats, the establishment of a developed BTB is necessary for type A spermatogonia differentiation and the initiation of the meiosis cycle (Mok et al., 2011a). During testis maturation in rats, integral TJ proteins occludin and claudin-11 were colocalized with the scaffold protein ZO-1 at the BTB after 25–30 days *postpartum* (dpp) (Figure 2). GJ proteins and basal ES proteins were mapped as indicators of BTB assembly (Mok et al., 2011b).

During development and tissular morphogenesis, CAR is present at high levels, mediating cell adhesion (Mirza et al., 2007). Few studies using different animal models targeted the IF position of CAR in the seminiferous epithelium during testis maturation. Its presence (Wang et al., 2007) or absence (Mirza et al., 2007) in adult Sertoli cells was discussed and needs further and accurate microscopy studies.

The BTB assembly during puberty was studied in 14–20 dpp using TEM and permeability tracers, showing an association between the absence of the BTB and the apoptosis of zygotene-pachytene spermatocytes (Morales et al., 2007).

Occludin was targeted in embryonic and developing mouse testis showing a diffuse localization in embryonic cords and a reorganization at the basal junction of postnatal prepuberal individuals, showing the initial TJ layout (Cyr et al., 1999).

6. BTB and transcellular germ cell migration

The transfer of spermatocytes from one compartment (basal) of the seminiferous epithelium to another (adluminal) is necessary for germ cell maturation. In the adluminal compartment, meiosis is completed, and spermatids formation is followed by spermatid differentiation and spermatozoa release (Russell, 1977; Nishimura and L’Hernault, 2017). The progression of preleptotene/leptotene spermatocytes across the BTB (stage VIII in rats) is dynamic as spermatocytes penetrate through Sertoli cells TJ, along with an extensive restructuring of the BTB (Wang and Cheng, 2007; Mruk and Cheng, 2015). This event is crucial because BTB integrity cannot be disrupted. The old BTB above the preleptotene spermatocytes will be dismantled, but only after a new barrier is assembled behind. The 3-dimensional organization of some adhesion complexes was visualized in confocal studies of claudin-11 and claudin-3 during preleptotene spermatocyte movement across the BTB. Migrating cyst conformation was also verified by specific markers like testis expressed 14, intercellular bridge forming factor (TEX14) and F-actin. The moving cell syncytium is enclosed within TJ during while crossing the BTB (Smith and Braun, 2012). Under electron microscopy, preleptotene spermatocyte was observed between TJ after detaching from the basal lamina (Yan et al., 2008).

7. BTB and the controversy about the intermediate compartment

Morphological studies reveal a third compartment in the seminiferous epithelium (Russell, 1978). At the time germ cells cross the BTB, preleptotene/leptotene spermatocytes cannot be defined within either the basal or the adluminal (apical) compartment. Instead, there is an intermediate between them, sealed above and below migrating cells (Yazama, 2008). In the boar mature testis, the intermediate compartment was identified using TEM during spermatocyte migration (Yazama, 2008).

8. Junction assembly and dismantling in seasonal breeders

Testosterone regulates cell adhesion proteins in the seminiferous tubules (Meng et al., 2005; McCabe et al., 2010). In seasonal breeders, testosterone controls the activity of the spermatogenic epithelium, whereby cell sloughing with disorganization of protein complexes at the adherens junctions is observed during the inactive season. A comparable regression occurs in typical models of experimental androgen reduction (Haverfield et al., 2014). In animals with seasonal spermatogenesis, cell junction proteins were localized in the regressing testes of several species as the armadillo and the Iberian mole (Dadhich et al., 2013; Luaces et al., 2014).

Many works of mammalian species with seasonal spermatogenesis analyzed the function of the BTB with ultrastructural studies (Pelletier, 1986; Morales and Cavicchia, 1993; Pelletier, 2011a) and immunolocalization of different proteins (Massoud et al., 2021). Freeze-facture and TEM studies of the inter-Sertoli cell junctions were done in the viscacha (*Lagostomus maximus*), a seasonal reproducer that presents no BTB competence during the inactive period, as confirmed using permeability tracers (Morales and Cavicchia, 1993).

In the large hairy armadillo (*Chaetophractus villosus*), different proteins of the Sertoli cell junctions were analyzed during the active phase of spermatogenesis over the different stages of the seminiferous epithelium and in the regressive testis phase (Luaces et al., 2014; Merico et al., 2019). Co-localization of N-cadherin and β-catenin was observed at the base during the active phase, and a decrease and diffused localization in the staining pattern was shown during testis regression. Two kinases, FAK and c-Src, that play a key role in regulating BTB. dynamics were detected by IF at the basal portion of the seminiferous epithelium, with strong signals during the active phase of the testis with respect to the inactive and regressing testis. The phosphorylated forms of both kinases were not detected by IF in the basal compartment (Merico et al., 2019). In another seasonal breeder, the Iberian mole (*Talpa occidentalis*), structural markers of the BTB, like claudin-11, Cx43, E-cadherin, and N-cadherin, were studied. BTB was compromised after testis regression, as shown using permeability tracers and inferred by TEM (Dadhich et al., 2013). In the greater white-toothed shrew (*Crocidura russula*), claudin-11 was mapped during the active and regression phases of spermatogenesis (Massoud et al., 2014). BTB markers were studied in the testis of another species with the seasonal arrest of the spermatogenesis, the Egyptian long-eared hedgehog (*Hemiechinus auritus*), with a disruption of tight and gap junctions as shown by claudin-11 and Cx43, respectively. Adherens junction molecules, N-cadherin and β-catenin, were also targeted by IF (Massoud et al., 2018).

9. Junction assembly and dismantling in pathologies

In biopsies of patients with testicular carcinoma *in situ*, studies showed impaired development of Sertoli cell junctions and BTB functional integrity loss with changes in the distribution pattern of ZO-1 and ZO-2 (Fink et al., 2006). The role and localization of specific TJ molecules was studied during puberty in a cryptorchid rat model, claudin-11 staining pattern was characterized by an altered distribution of this integral protein, with claudin-11 stain parallel to the basal membrane in control vs. vertical stain in non-descended testes (Kato et al., 2020, Figure 3). Spontaneous autoimmune orchitis was studied in the mink (Mustela vison) and Cx46 distribution was examined in the seminiferous epithelium of pathological testis (Pelletier et al., 2011b; Pelletier et al., 2015).

**FIGURE 3:**
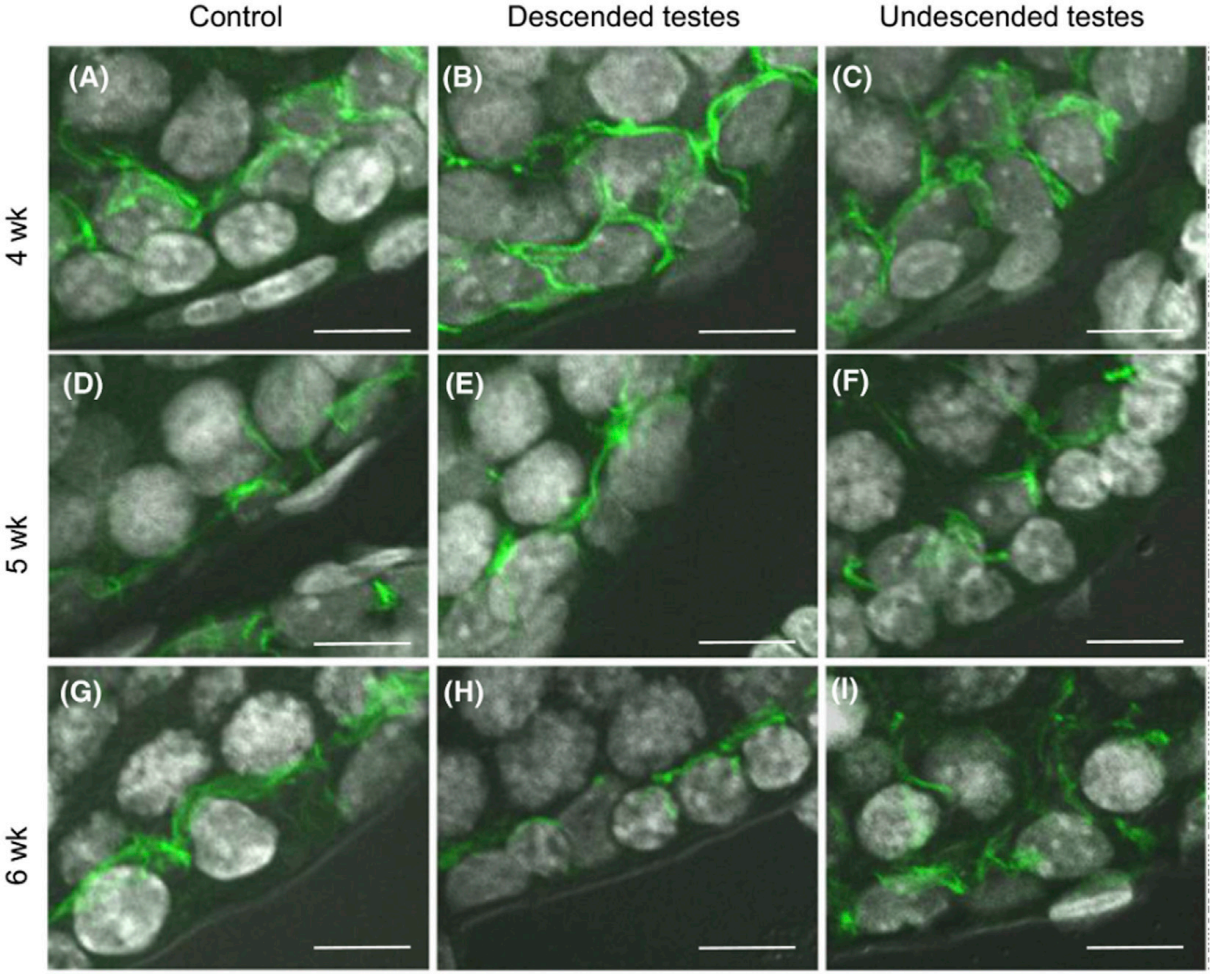
Blood-testis barrier morphology in a cryptorchid rat model. Claudin-11 (green) immunolocalization in rat testes at 4–6 weeks *postpartum*. After 5 weeks *postpartum*, claudin-11 staining was redistributed in control testes and descended testes, from vertical to parallel with respect to the basement membrane. Staining pattern was unaltered in the undescended testes, remaining vertical to the basement membrane. Scale bar = 10 μm. Figure reproduced with permission from Kato et al. (2020).

Infertility is associated with diet-induced obesity in animal models characterized by serum hyperlipidemia and hyperleptinemia, as in metabolic syndrome (Fan et al., 2015). Altered cholesterol homeostasis in Sertoli cells should disturb BTB function due to regulating the endocytosis of junctions involved in the BTB seal. In rabbits fed with a 2% cholesterol-enriched diet, the identification of ZO-1 and occludin suggested a disruption of the BTB using a permeability assay (Morgan et al., 2014). In the HFD mice model, a dysfunctional BTB was concurrent with altered relevant biomarkers of the BTB function. Both TEM and permeability assay showed that BTB integrity was compromised in this model (Fan et al., 2015). In the mouse testis, IF localization of Cx43, Cx46 and Cx50 was analyzed using models of diabetes and obesity (Pelletier et al., 2018; Pelletier et al., 2020).

10. BTB and contraceptives

Non-hormonal alternatives are desirable for male contraception in order to minimize side effects. Several works analyzed compounds to induce post-meiotic germ cell depletion from the testis as contraceptives (Mao et al., 2019b). Drug delivery at the target is challenging since the BTB might block molecules’ passage (Cheng and Mruk, 2002; Cheng and Mruk, 2012). Adjudin affects Sertoli-germ cell adhesion, producing germ cell shedding and temporal infertility, depleting meiotic and postmeiotic cells from the testis (Yan and Cheng, 2005; Mruk and Cheng, 2010). The *in vivo* murine model of adjudin reveals changes in Cx43 stain intensity in treated testes (Li et al., 2009).

11. BTB and toxicants

The effect of environmental contaminants on BTB alteration has been confirmed (Zhu et al., 2020). Sobarzo et al. (2009) demonstrated that Di (2-ethylhexyl) phthalate induces a delocalization of claudin-11 in rat testis.

One of the most studied environmental pollutants with detrimental effects on testis function is Cadmium, a heavy metal, as in cadmium chloride (CdCl2). Humans are exposed to Cd through pollutants in the air, drinking water, and food, and Cd impairs Sertoli cell development during fetal and neonatal phases (Zhu et al., 2020). On gestational day 12, a single Cd intraperitoneal injection up to 1.0 mg/kg to 64-day-old Sprague-Dawley dams dose-dependently impaired fetal testis production of testosterone, reduced fetal Leydig cell numbers, and downregulated protein expression in Leydig and Sertoli cells (Li X et al., 2018). The CdCl_2_ model is widely used to study the TJ of Sertoli cells. This compound is used to study Sertoli cell TJ dynamics, damages the BTB, and has irreversible effects on the testis (Zhu et al., 2020). BTB markers were analyzed in rodent models exposed to cadmium and disruption of the barrier was observed by TEM and IF of ZO-1, occludin, N-cadherin, and β-catenin (Wong et al., 2004; Wong et al., 2005). Cadmium also affects BTB by reducing occludin expression in mice (Zhou et al., 2022) and altering the localization of Cx43 and occludin in rats. A recent study shows that a traditional Chinese medicine prevented Cd-induced BTB damage opposing to Cd-effects on the PI3K/Akt/Rictor signaling pathway (RICTOR, the rapamycin-insensitive companion of mTOR, is the scaffold protein for substrate binding to mTORC2, which promotes BTB integrity) (Shen et al., 2023).

Microplastics (MPs), emerging pollutant particles smaller than 5 mm, have been reported to induce testicular disorder in mammals (Wen et al., 2022). In “*in vitro*” studies, 0.5 μm–10 μm MPs were internalized by the germ, Leydig, and Sertoli cells (Jin et al., 2021), disrupting BTB and inducing spermatocyte apoptosis in testis via inflammation and oxidative stress (Wei et al., 2021; Yin et al., 2021). Likewise, *in vivo*, MPs affected sperm count, motility and abnormality rate, and sex hormones level (Xie et al., 2020). Polystyrene microplastics disrupted BTB integrity, as evidenced by actin localization in mice (Wei et al., 2021). Oral treatment with a polystyrene MPs and cadmium combination was harmful to the testicular activity of sexually mature rats, as evidenced by histological and biomolecular alterations. MPs accumulated in the seminiferous epithelium, induced oxidative stress, and affected sperm quality. BTB cytoarchitecture of the blood-testis barrier was compromised, as revealed by lower levels of structural occludin, Van Gogh-like protein 2, and connexin 43 (Hassine et al., 2023). The simultaneous administration of microplastics and cadmium affected the cytoarchitecture of testicular cells and normal spermatogenesis, impairing expression and localization of two proteins—DAAM1 and PREP—involved in actin- and microtubule-associated processes, during germ cells differentiation into spermatozoa (Venditti et al., 2023).

12. Permeability tracers and the BTB

Several methods are available to determine BTB permeability rate *in situ* using electron or light microscopy. Lanthanum nitrate and horseradish peroxidase are the most widely used in TEM. Lanthanum has the disadvantage of forming precipitates responsible for false positives and unreliable staining (Mann et al., 2003). Horseradish peroxidase infusion through the vascular system showed no tracer precipitates and gives more reliable results (Pelletier and Byers, 1992; Lustig et al., 2000). Different tracers tested in testosterone deprivation models *in vivo* showed varying permeability rates in relation to their molecular weight (Haverfield et al., 2014). During spermatogenic re-initiation, small, medium, and large-sized tracers were used to map the extension of the BTB permeation. The magnitude of permeation was associated with a particular germ cell, according to spermatid maturation after spermatogenesis’ re-initiation (Haverfield et al., 2014).

13. Unexplored issues in microscopy and new technologies

The study of BTB structure calls for a technical upgrade, using new technological advances to review previous, classic, and/or established concepts.

Super-resolution microscopy emerged as a powerful tool for the study of cellular biology in the last two decades, allowing visualization of molecular structures below the visible light diffraction limit (around 250 nm). Super-resolution microscopy accuracy allows molecular localization within the nanoscale range, which is required to understand the significant diversity of protein interactions in the BTB *in vitro* and *in situ* (Zhang et al., 2013; Adams et al., 2018).

Three-dimensional structures like cells, organelles, and the ultrastructure of the cellular membrane can be examined using confocal microscopy (Du et al., 2013) and Electron Tomography (ET) (Lucić et al., 2005). The intricate membrane and subcellular structures like the Golgi complex, endosomes, and lysosomes can be studied with ET. BTB cells display active migratory movements, with cellular processes on spermatids and Sertoli cells. Some Sertoli cell processes penetrate spermatocytes cytoplasm, showing an active and coordinated migration. ET is also used to study cell pseudopodia, allowing us to understand the complex nature of the intracellular membrane structure in the BTB (Pelletier, 2011a).

Fine-resolution techniques have been used to analyze Sertoli cell organelles in rat testis. Using Electron tomography, the 3D aspect of tubulobulbar complexes was described (Lyon et al., 2017). Basal ES elements in the BTB, like the stromal interacting molecule 1 (STIM1), a sensor in the ER membrane, were also viewed using confocal microscopy (Lyon et al., 2017).

Also, using Fluorescent Labeling of Abundant Reactive Entities (FLARE) and expansion super-resolution microscopy, spermatid development was imaged showing specific carbohydrate patterns (Mao et al., 2020). In contrast to antibody labeling, FLARE enables staining thick specimens with fluorescent dyes for intense covalent labeling in super-resolution microscopy (and cleared tissue microscopy) (Mao et al., 2020).

## Concluding remarks

The blood-testis barrier has a central role in spermatogenesis regulation. So far, the classical ultrastructural and IF research framework has supported BTB functional morphology. As observed in a standard cross-section of the testis, there’s a great diversity of cell associations that occur between cells. During spermatogenesis, BTB needs to keep a safe and isolated environment for postmeiotic germ cell maturation. Because of its complex structure, the BTB can be fully understood only considering the whole seminiferous epithelium *in situ*. Imaging the dynamic nature of the junctions that seal the adluminal space is a challenge for morphological studies. Therefore, visualization of specific structures and interactions of the junctions at a high spatial resolution is necessary to improve the actual knowledge of the BTB. Further studies using microscopic techniques like electron tomography and confocal microscopy, which ponder the three-dimensional junction structures, are yet to be done. The junctions at the BTB are one of the largest obstacles to male contraception, since many drugs cannot reach the adluminal compartment. Addressing BTB functional morphology issues will help to understand how it is regulated and design new strategies for male fertility control and contraception.

## References

[B1] Adams A. Sriram A. Vogl A. W. (2018). Internalization of intact intercellular junctions in the testis by clathrin/actin‐mediated endocytic structures: Tubulobulbar complexes. Anat. Rec. 301 (12), 2080–2085. 10.1002/ar.23963 30312540

[B2] Aumüller G. Schulze C. Viebahn C. (1992). Intermediate filaments in Sertoli cells. Microsc. Res. Tech. 20 (1), 50–72. 10.1002/jemt.1070200107 1377059

[B3] Budipitojo T. Sasaki M. Nurliani A. Ariana Mulyani G. T. Kitamura N. (2018). An immunohistochemical study of the cytoskeletal proteins in the testis of the sunda porcupine (Hystrix javanica). Mammal. Study 43 (2), 117–123. 10.3106/ms2017-0052

[B4] Byers S. Graham R. Dai H. N. Hoxter B. (1991). Development of Sertoli cell junctional specializations and the distribution of the tight‐junction‐associated protein ZO‐1 in the mouse testis. Am. J. Anat. 191 (1), 35–47. 10.1002/aja.1001910104 2063808

[B5] Carette D. Weider K. Gilleron J. Giese S. Dompierre J. Bergmann M. (2010). Major involvement of connexin 43 in seminiferous epithelial junction dynamics and male fertility. Dev. Biol. 346 (1), 54–67. 10.1016/j.ydbio.2010.07.014 20655897

[B6] Cavicchia J. C. Sacerdote F. L. (1991). Correlation between blood‐testis barrier development and onset of the first spermatogenic wave in normal and in busulfan‐treated rats: A lanthanum and freeze‐fracture study. Anat. Rec. 230 (3), 361–368. 10.1002/ar.1092300309 1867410

[B7] Cavicchia J. C. Sacerdote F. L. (1988). Topography of the rat blood-testis barrier after intratubular administration of intercellular tracers. Tissue Cell 20 (4), 577–586. 10.1016/0040-8166(88)90059-6 3238690

[B8] Cheng C. Y. Mruk D. D. (2002). Cell junction dynamics in the testis: Sertoli-germ cell interactions and male contraceptive development. Physiol. Rev. 82 (4), 825–874. 10.1152/physrev.00009.2002 12270945

[B9] Cheng C. Y. Mruk D. D. (2012). The blood-testis barrier and its implications for male contraception. Pharmacol. Rev. 64 (1), 16–64. 10.1124/pr.110.002790 22039149 PMC3250082

[B10] Chihara M. Otsuka S. Ichii O. Hashimoto Y. Kon Y. (2010). Molecular dynamics of the blood–testis barrier components during murine spermatogenesis. Mol. Reprod. Dev. 77 (7), 630–639. 10.1002/mrd.21200 20578065

[B11] Cyr D. G. Hermo L. Egenberger N. Mertineit C. Trasler J. M. Laird D. W. (1999). Cellular immunolocalization of occludin during embryonic and postnatal development of the mouse testis and epididymis. Endocrinology 140 (8), 3815–3825. 10.1210/endo.140.8.6903 10433243

[B12] Dadhich R. K. Barrionuevo F. J. Real F. M. Lupiañez D. G. Ortega E. Burgos M. (2013). Identification of live germ-cell desquamation as a major mechanism of seasonal testis regression in mammals: A study in the iberian mole (*Talpa occidentalis*). Biol. Reprod. 88 (4), 101–1. 10.1095/biolreprod.112.106708 23515671

[B13] Davidoff M. S. Middendorff R. Pusch W. Müller D. Wichers S. Holstein A. F. (1999). Sertoli and Leydig cells of the human testis express neurofilament triplet proteins. Histochem. Cell Biol. 111 (3), 173–187. 10.1007/s004180050347 10094414

[B14] Domke L. M. Rickelt S. Dörflinger Y. Kuhn C. Winter-Simanowski S. Zimbelmann R. (2014). The cell-cell junctions of mammalian testes: I. The adhering junctions of the seminiferous epithelium represent special differentiation structures. Cell Tissue Res. 357 (3), 645–665. 10.1007/s00441-014-1906-9 24907851 PMC4148596

[B15] Du M. Young J. N. De Asis M. Cipollone J. Roskelley C. Takai Y. (2013). A novel subcellular machine contributes to basal junction remodeling in the seminiferous epithelium. Biol. Reprod. 88 (3), 60–61. 10.1095/biolreprod.112.104851 23303684

[B16] Dym M. Cavicchia J. C. (1977). Further observations on the blood-testis barrier in monkeys. Biol. Reprod. 17 (3), 390–403. 10.1095/biolreprod17.3.390 409441

[B17] Dym M. Fawcett D. W. (1970). The blood-testis barrier in the rat and the physiological compartmentation of the seminiferous epithelium. Biol. Reprod. 3 (3), 308–326. 10.1093/biolreprod/3.3.308 4108372

[B18] Fan Y. Liu Y. Xue K. Gu G. Fan W. Xu Y. (2015). Diet-induced obesity in male C57BL/6 mice decreases fertility as a consequence of disrupted blood-testis barrier. PloS one 10 (4), e0120775. 10.1371/journal.pone.0120775 25886196 PMC4401673

[B19] Fink C. Weigel R. Hembes T. Lauke-Wettwer H. Kliesch S. Bergmann M. (2006). Altered expression of ZO-1 and ZO-2 in Sertoli cells and loss of blood-testis barrier integrity in testicular carcinoma *in situ* . Neoplasia 8 (12), 1019–1027. 10.1593/neo.06559 17217619 PMC1783719

[B115] França L. R. Hess R. A. Dufour J. M. Hofmann M. C. Griswold M. D. (2016). The sertoli cell: one hundred fifty years of beauty and plasticity. Andrology 4 (2), 189–212. 10.1111/andr.12165 26846984 PMC5461925

[B20] Guttman J. A. Mulholland D. J. Vogl A. W. (1999). Plectin is concentrated at intercellular junctions and at the nuclear surface in morphologically differentiated rat Sertoli cells. Anat. Rec. 254 (3), 418–428. 10.1002/(SICI)1097-0185(19990301)254:3<418::AID-AR13>3.0.CO;2-C 10096674

[B21] Hassine M. B. H. Venditti M. Rhouma M. B. Minucci S. Messaoudi I. (2023). Combined effect of polystyrene microplastics and cadmium on rat blood-testis barrier integrity and sperm quality. Environ. Sci. Pollut. Res. Int. 30 (19), 56700–56712. 10.1007/s11356-023-26429-z 36928700

[B22] Haverfield J. T. Meachem S. J. Nicholls P. K. Rainczuk K. E. Simpson E. R. Stanton P. G. (2014). Differential permeability of the blood-testis barrier during reinitiation of spermatogenesis in adult male rats. Endocrinology 155 (3), 1131–1144. 10.1210/en.2013-1878 24424039

[B23] Hellani A. Ji J. Mauduit C. Deschildre C. Tabone E. Benahmed M. (2000). Developmental and hormonal regulation of the expression of oligodendrocyte-specific protein/claudin 11 in mouse testis. Endocrinology 141 (8), 3012–3019. 10.1210/endo.141.8.7625 10919290

[B24] Hogarth C. A. Griswold M. D. (2013). Immunohistochemical approaches for the study of spermatogenesis. Methods Mol. Biol. 927, 309–320. 10.1007/978-1-62703-038-0_28 22992925

[B117] Jiang X. H. Bukhari I. Zheng W. Yin S. Wang Z. Cooke H. J. (2014). Blood-testis barrier and spermatogenesis: lessons from genetically-modified mice. Asian J. Androl. 16 (4), 572–580. 10.4103/1008-682X.125401 24713828 PMC4104086

[B25] Jin H. Ma T. Sha X. Liu Z. Zhou Y. Meng X. (2021). Polystyrene microplastics induced male reproductive toxicity in mice. J. Hazard Mater 401, 123430. PMID: 32659591. 10.1016/j.jhazmat.2020.123430 32659591

[B26] Johnson K. J. Boekelheide K. (2002b). Dynamic testicular adhesion junctions are immunologically unique. II. Localization of classic cadherins in rat testis. Biol. Reprod. 66 (4), 992–1000. 10.1095/biolreprod66.4.992 11906918

[B27] Johnson K. J. Boekelheide K. (2002a). Dynamic testicular adhesion junctions are immunologically unique. I. Localization of p120 catenin in rat testis. Biol. reproduction 66 (4), 983–991. 10.1095/biolreprod66.4.983 11906917

[B28] Kan F. W. Lin Y. (1995). Immunogold localization of actin in the testis and exocrine pancreas: Spatial relationship with tight junctional strands. Microsc. Res. Tech. 31 (2), 128–140. 10.1002/jemt.1070310205 7655087

[B29] Kato T. Mizuno K. Nishio H. Moritoki Y. Kamisawa H. Kurokawa S. (2020). Disorganization of claudin-11 and dysfunction of the blood-testis barrier during puberty in a cryptorchid rat model. Andrology 8 (5), 1398–1408. 10.1111/andr.12788 32196966

[B30] Leblond C. P. Clermont Y. (1952). Definition of the stages of the cycle of the seminiferous epithelium in the rat. Ann. N. Y. Acad. Sci. 55, 548–573. 10.1111/J.1749-6632.1952.TB26576.X 13139144

[B31] Li L. Mao B. Yan M. Wu S. Ge R. Lian Q. (2019). Planar cell polarity protein Dishevelled 3 (Dvl3) regulates ectoplasmic specialization (ES) dynamics in the testis through changes in cytoskeletal organization. Cell Death Dis. 10, 194. 10.1038/s41419-019-1394-7 30808893 PMC6391420

[B32] Li M. W. Mruk D. D. Lee W. M. Cheng C. Y. (2009). Connexin 43 and plakophilin-2 as a protein complex that regulates blood–testis barrier dynamics. PNAS 106 (25), 10213–10218. 10.1073/pnas.0901700106 19509333 PMC2700929

[B33] Li S. Y. Mruk D. D. Cheng C. Y. (2013). Focal adhesion kinase is a regulator of F-actin dynamics: New insights from studies in the testis. Spermatogenesis 3 (3), e25385. 10.4161/spmg.25385 24381802 PMC3861170

[B34] Li X. Liu J. Wu S. Zheng W. Li H. Bao S. (2018). *In utero* single low-dose exposure of cadmium induces rat fetal Leydig cell dysfunction. Chemosphere 194, 57–66. 10.1016/j.chemosphere.2017.11.159 29197250

[B35] Li X. Y. Zhang Y. Wang X. X. Jin C. Wang Y. Q. Sun T. C. (2018). Regulation of blood‐testis barrier assembly *in vivo* by germ cells. FASEB J. 32 (3), 1653–1664. 10.1096/fj.201700681R 29183964 PMC6137450

[B36] Lie P. P. Mruk D. D. Mok K. W. Su L. Lee W. M. Cheng C. Y. (2012). Focal adhesion kinase-Tyr407 and-Tyr397 exhibit antagonistic effects on blood–testis barrier dynamics in the rat. PNAS 109 (31), 12562–12567. 10.1073/pnas.1202316109 22797892 PMC3412022

[B37] Liu S. Li H. Wu S. Li L. Ge R. Cheng C. Y. (2020). NC1‐peptide regulates spermatogenesis through changes in cytoskeletal organization mediated by EB1. FASEB J. 34 (2), 3105–3128. 10.1096/fj.201901968RR 31909540

[B38] Luaces J. P. Rossi L. F. Sciurano R. B. Rebuzzini P. Merico V. Zuccotti M. (2014). Loss of Sertoli-germ cell adhesion determines the rapid germ cell elimination during the seasonal regression of the seminiferous epithelium of the large hairy armadillo *Chaetophractus villosus* . Biol. Reprod. 90 (3), 48–51. 10.1095/biolreprod.113.113118 24451984

[B39] Lucić V. Förster F. Baumeister W. (2005). Structural studies by electron tomography: From cells to molecules. Annu. Rev. Biochem. 74, 833–865. 10.1146/annurev.biochem.73.011303.074112 15952904

[B40] Lui W. Y. Mruk D. Lee W. M. Cheng C. Y. (2003). Sertoli cell tight junction dynamics: Their regulation during spermatogenesis. Biol. Reprod. 68 (4), 1087–1097. 10.1095/biolreprod.102.010371 12606453

[B41] Lustig L. Denduchis B. Ponzio R. Lauzon M. Pelletier R. M. (2000). Passive immunization with anti-laminin immunoglobulin G modifies the integrity of the seminiferous epithelium and induces arrest of spermatogenesis in the Guinea pig. Biol. Reprod. 62 (6), 1505–1514. 10.1095/biolreprod62.6.1505 10819750

[B42] Lyon K. Adams A. Piva M. Asghari P. Moore E. D. Vogl A. W. (2017). Ca2+ signaling machinery is present at intercellular junctions and structures associated with junction turnover in rat Sertoli cells. Biol. Reprod. 96 (6), 1288–1302. 10.1093/biolre/iox042 28486663

[B43] Maekawa M. Toyama Y. Yasuda M. Yagi T. Yuasa S. (2002). Fyn tyrosine kinase in Sertoli cells is involved in mouse spermatogenesis. Biol. Reprod. 66 (1), 211–221. 10.1095/biolreprod66.1.211 11751285

[B44] Mann M. C. Friess A. E. Stoffel M. H. (2003). Blood-tissue barriers in the male reproductive tract of the dog: A morphological study using lanthanum nitrate as an electron-opaque tracer. Cells Tissues Organs 174 (4), 162–169. 10.1159/000072719 14504427

[B45] Mao B. P. Li L. Ge R. Li C. Wong C. K. Silvestrini B. (2019a). CAMSAP2 is a microtubule minus-end targeting protein that regulates BTB dynamics through cytoskeletal organization. Endocrinology 160 (6), 1448–1467. 10.1210/en.2018-01097 30994903 PMC6530524

[B46] Mao B. P. Li L. Yan M. Ge R. Lian Q. Cheng C. Y. (2019b). Regulation of BTB dynamics in spermatogenesis-insights from the adjudin model. Toxicol. Sci. 172 (1), 75–88. 10.1093/toxsci/kfz180 31397872 PMC6813747

[B47] Mao C. Lee M. Y. Jhan J. R. Halpern A. R. Woodworth M. A. Glaser A. K. (2020). Feature-rich covalent stains for super-resolution and cleared tissue fluorescence microscopy. Sci. Adv. 6 (22), eaba4542. eaba4542. 10.1126/sciadv.aba4542 32518827 PMC7253160

[B48] Massoud D. Barrionuevo F. J. Ortega E. Burgos M. Jiménez R. (2014). The testis of greater white-toothed shrew crocidura russula in southern European populations: A case of adaptive lack of seasonal involution? J. Exp. Zool. B Mol. 322 (5), 304–315. 10.1002/jez.b.22582 24895181

[B49] Massoud D. Lao-Perez M. Hurtado A. Abdo W. Palomino-Morales R. Carmona F. D. (2018). Germ cell desquamation-based testis regression in a seasonal breeder, the Egyptian long-eared hedgehog, Hemiechinus auritus. PLoS One 13 (10), e0204851. 10.1371/journal.pone.0204851 30286149 PMC6171879

[B50] Massoud D. Lao-Pérez M. Ortega E. Burgos M. Jiménez R. Barrionuevo F. J. (2021). Divergent seasonal reproductive patterns in syntopic populations of two murine species in southern Spain, *Mus spretus* and *Apodemus sylvaticus* . Animals 11 (2), 243. 10.3390/ani11020243 33498171 PMC7908971

[B51] McCabe M. J. Tarulli G. A. Meachem S. J. Robertson D. M. Smooker P. M. Stanton P. G. (2010). Gonadotropins regulate rat testicular tight junctions *in vivo* . Endocrinology 151 (6), 2911–2922. 10.1210/en.2009-1278 20357222 PMC2875820

[B52] Meng J. Holdcraft R. W. Shima J. E. Griswold M. D. Braun R. E. (2005). Androgens regulate the permeability of the blood–testis barrier. PNAS 102 (46), 16696–16700. 10.1073/pnas.0506084102 16275920 PMC1283811

[B53] Merico V. Luaces J. P. Rossi L. F. Rebuzzini P. Merani M. S. Zuccotti M. (2019). Sertoli–immature spermatids disengagement during testis regression in the armadillo. Reproduction 157 (1), 27–42. 10.1530/REP-18-0006 30394707

[B54] Mirza M. Petersen C. Nordqvist K. Sollerbrant K. (2007). Coxsackievirus and adenovirus receptor is up-regulated in migratory germ cells during passage of the blood-testis barrier. Endocrinology 148 (11), 5459–5469. 10.1210/en.2007-0359 17690169

[B55] Mok K. W. Mruk D. D. Lee W. M. Cheng C. Y. (2011b). A study to assess the assembly of a functional blood-testis barrier in developing rat testes. Spermatogenesis 1 (3), 270–280. 10.4161/spmg.1.3.17998 22319674 PMC3271668

[B56] Mok K. W. Mruk D. D. Lee W. M. Cheng C. Y. (2011a). Spermatogonial stem cells alone are not sufficient to re‐initiate spermatogenesis in the rat testis following adjudin‐induced infertility. Int. J. Androl. 35 (1), 86–101. 10.1111/j.1365-2605.2011.01183.x 21696392 PMC3457811

[B57] Morales A. Mohamed F. Cavicchia J. C. (2007). Apoptosis and blood-testis barrier during the first spermatogenic wave in the pubertal rat. Anat. Rec. Hob. 290 (2), 206–214. 10.1002/ar.20417 17441213

[B58] Morales A. Cavicchia J. C. (1993). Seasonal changes of the blood‐testis barrier in viscacha (*Lagostomus maximus* maximus): A freeze‐fracture and lanthanum tracer study. Anat. Rec. 236 (3), 459–464. 10.1002/ar.1092360306 8363051

[B59] Morgan D. H. Ghribi O. Hui L. Geiger J. D. Chen X. (2014). Cholesterol-enriched diet disrupts the blood-testis barrier in rabbits. Am. J. Physiol. Endocrinol. 307 (12), E1125–E1130. 10.1152/ajpendo.00416.2014 PMC4269676 25336525

[B60] Morita K. Sasaki H. Fujimoto K. Furuse M. Tsukita S. (1999). Claudin-11/OSP-based tight junctions of myelin sheaths in brain and Sertoli cells in testis. J. Cell Biol. 145 (3), 579–588. 10.1083/jcb.145.3.579 10225958 PMC2185072

[B61] Morrow C. M. Tyagi G. Simon L. Carnes K. Murphy K. M. Cooke P. S. (2009). Claudin 5 expression in mouse seminiferous epithelium is dependent upon the transcription factor ets variant 5 and contributes to blood-testis barrier function. Biol. Reprod. 81 (5), 871–879. 10.1095/biolreprod.109.077040 19571261 PMC2770019

[B62] Mruk D. D. Cheng C. Y. (2010). Delivering non-hormonal contraceptives to men: Advances and obstacles. Trends Biotechnol. 26 (2), 90–99. 10.1016/j.tibtech.2007.10.009 PMC4035909 18191256

[B63] Mruk D. D. Cheng C. Y. (2011). An *in vitro* system to study Sertoli cell blood-testis barrier dynamics. Methods Mol. Biol. 763, 237–252. 10.1007/978-1-61779-191-8_16 21874456 PMC4138974

[B64] Mruk D. D. Cheng C. Y. (2012). In search of suitable *in vitro* models to study germ cell movement across the blood-testis barrier. Spermatogenesis 2 (1), 6–10. 10.4161/spmg.19878 22553485 PMC3341247

[B65] Mruk D. D. Cheng C. Y. (2004). Sertoli-Sertoli and Sertoli-germ cell interactions and their significance in germ cell movement in the seminiferous epithelium during spermatogenesis. Endocr. Rev. 25 (5), 747–806. 10.1210/er.2003-0022 15466940

[B66] Mruk D. D. Cheng C. Y. (2015). The mammalian blood-testis barrier: Its biology and regulation. Endocr. Rev. 36 (5), 564–591. 10.1210/er.2014-1101 26357922 PMC4591527

[B67] Mruk D. D. Cheng C. Y. (2010). Tight junctions in the testis: New perspectives. Philos. Trans. R. Soc. Lond. B. Biol. Sci. 365 (1546), 1621–1635. 10.1098/rstb.2010.0010 20403874 PMC2871926

[B68] Mulholland D. J. Dedhar S. Vogl W. A. (2001). Rat seminiferous epithelium contains a unique junction (ectoplasmic specialization) with signaling properties both of cell/cell and cell/matrix junctions. Biol. Reprod. 64 (1), 396–407. 10.1095/biolreprod64.1.396 11133699

[B69] Nagano T. Suzuki F. (1976). The postnatal development of the junctional complexes of the mouse Sertoli cells as revealed by freeze‐fracture. Anat. Rec. 185 (4), 403–417. 10.1002/ar.1091850403 970659

[B70] Nishimura H. L’Hernault S. W. (2017). Spermatogenes. Curr. Biol. 27 (18), R988–R994. 10.1016/j.cub.2017.07.067 28950090

[B71] Nollet F. Kools P. van Roy F. (2000). Phylogenetic analysis of the cadherin superfamily allows identification of six major subfamilies besides several solitary members. J. Mol. Biol. 299 (3), 551–572. 10.1006/jmbi.2000.3777 10835267

[B72] Pelletier R. M. Akpovi C. D. Chen L. Day R. Vitale M. L. (2011b). CX43 expression, phosphorylation, and distribution in the normal and autoimmune orchitic testis with a look at gap junctions joining germ cell to germ cell. Am. J. Physiol. Regul. Integr. Comp. Physiol. 300 (1), R121–R139. 10.1152/ajpregu.00500.2010 20962206

[B73] Pelletier R. M. Akpovi C. D. Chen L. Kumar N. M. Vitale M. L. (2015). Complementary expression and phosphorylation of Cx46 and Cx50 during development and following gene deletion in mouse and in normal and orchitic mink testes. Am. J. Physiol. Regul. Integr. Comp. Physiol. 309 (3), R255–R276. 10.1152/ajpregu.00152.2015 26017495 PMC4525330

[B74] Pelletier R. M. Akpovi C. D. Chen L. Vitale M. L. (2018). Cholesterol metabolism and Cx43, Cx46, and Cx50 gap junction protein expression and localization in normal and diabetic and obese ob/ob and db/db mouse testes. Am. J. Physiol. Endocrinol. 314 (1), E21–E38. 10.1152/ajpendo.00215.2017 PMC5866387 28851737

[B75] Pelletier R. M. Byers S. W. (1992). The blood‐testis barrier and Sertoli cell junctions: Structural considerations. Microsc. Res. Tech. 20 (1), 3–33. 10.1002/jemt.1070200104 1611148

[B76] Pelletier R. M. (1986). Cyclic formation and decay of the blood‐testis barrier in the mink (Mustela vison), a seasonal breeder. Am. J. Anat. 175 (1), 91–117. 10.1002/aja.1001750109 3953473

[B77] Pelletier R. M. Friend D. S. (1983). The sertoli cell junctional complex: Structure and permeability to filipin in the neonatal and adult Guinea pig. Am. J. Anat. 168 (2), 213–228. 10.1002/aja.1001680208 6650436

[B78] Pelletier R. M. Layeghkhavidaki H. Kumar N. M. Vitale M. L. (2020). Cx30. 2 deletion causes imbalances in testicular Cx43, Cx46, and Cx50 and insulin receptors. Reciprocally, diabetes/obesity alters Cx30. 2 in mouse testis. Am. J. Physiol. Regul. Integr. Comp. Physiol. 318 (6), R1078–R1090. 10.1152/ajpregu.00044.2020 32348681 PMC7311678

[B79] Pelletier R. M. (2011a). The blood-testis barrier: The junctional permeability, the proteins and the lipids. Prog. Histochem. Cytochem. 46 (2), 49–127. 10.1016/j.proghi.2011.05.001 21705043

[B80] Pointis G. Segretain D. (2005). Role of connexin-based gap junction channels in testis. Trends Endocrinol. Metab. 16 (7), 300–306. 10.1016/j.tem.2005.07.001 16054834

[B81] Ribeiro A. F. David‐Ferreira J. F. (1996). The inter‐sertoli cell tight junctions in germ cell‐free seminiferous tubules from prenatally irradiated rats: A freeze‐fracture study. Cell Biol. Int. 20 (7), 513–522. 10.1006/cbir.1996.0066 8931318

[B82] Risley M. S. Tan I. P. Roy C. Saez J. C. (1992). Cell-age-and stage-dependent distribution of connexin43 gap junctions in testes. J. Cell Sci. 103 (1), 81–96. 10.1242/jcs.103.1.81 1331136

[B83] Russell L. D. Peterson R. N. (1985). Sertoli cell junctions: Morphological and functional correlates. Int. Rev. Cytol. 94, 177–211. 10.1016/S0074-7696(08)60397-6 3894273

[B84] Russell L. D. (1978). The blood-testis barrier and its formation relative to spermatocyte maturation in the adult rat: A lanthanum tracer study. Anat. Rec. 190 (1), 99–111. 10.1002/ar.1091900109 626419

[B85] Russell L. (1977). Movement of spermatocytes from the basal to the adluminal compartment of the rat testis. Am. J. Anat. 148 (3), 313–328. 10.1002/aja.1001480303 857632

[B86] Schimenti K. J. Feuer S. K. Griffin L. B. Graham N. R. Bovet C. A. Hartford S. (2013). AKAP9 is essential for spermatogenesis and Sertoli cell maturation in mice. Genetics 194 (2), 447–457. 10.1534/genetics.113.150789 23608191 PMC3664854

[B87] Setchell B. P. Voglmayr J. K. Waites G. M. H. (1969). A blood-testis barrier restricting passage from blood into rete testis fluid but not into lymph. Physiol. J. 200 (1), 73–85. 10.1113/jphysiol.1969.sp008682 PMC1350418 4973530

[B88] Shen Y. You Y. Zhu K. Li G. Huang X. Chen D. (2023). The traditional Chinese medicine Qiangjing tablet prevents blood-testis barrier injury induced by CdCl2 through the PI3K/Akt/Rictor signaling pathway. Environ. Toxicol. 38 (3), 591–603. Epub 2022 Nov 12. PMID: 36370150. 10.1002/tox.23706 36370150

[B89] Show M. D. Anway M. D. Folmer J. S. Zirkin B. R. (2003). Reduced intratesticular testosterone concentration alters the polymerization state of the Sertoli cell intermediate filament cytoskeleton by degradation of vimentin. Endocrinology 144 (12), 5530–5536. 10.1210/en.2003-0735 12970161

[B90] Siu E. R. Wong E. W. Mruk D. D. Porto C. S. Cheng C. Y. (2009a). Focal adhesion kinase is a blood–testis barrier regulator. PNAS 106 (23), 9298–9303. 10.1073/pnas.0813113106 19470647 PMC2695058

[B91] Siu E. R. Wong E. W. Mruk D. D. Sze K. L. Porto C. S. Cheng C. Y. (2009b). An occludin-focal adhesion kinase protein complex at the blood-testis barrier: A study using the cadmium model. Endocrinology 150 (7), 3336–3344. 10.1210/en.2008-1741 19213829 PMC2703538

[B92] Smith B. E. Braun R. E. (2012). Germ cell migration across Sertoli cell tight junctions. Science 338 (6108), 798–802. 10.1126/science.1219969 22997133 PMC3694388

[B93] Sobarzo C. M. Lustig L. Ponzio R. Suescun M. O. Denduchis B. (2009). Effects of di (2‐ethylhexyl) phthalate on gap and tight junction protein expression in the testis of prepubertal rats. Microsc. Res. Tech. 72 (11), 868–877. 10.1002/jemt.20741 19526522

[B94] Stanton P. G. (2016). Regulation of the blood-testis barrier. Semin. Cell Dev. Biol. 59, 166–173. 10.1016/j.semcdb.2016.06.018 27353840

[B95] Su W. Wong E. W. Mruk D. D. Cheng C. Y. (2012). The Scribble/Lgl/Dlg polarity protein complex is a regulator of blood-testis barrier dynamics and spermatid polarity during spermatogenesis. Endocrinology 153 (12), 6041–6053. 10.1210/en.2012-1670 23038739 PMC3512062

[B96] Venditti M. Ben Hadj Hassine M. Messaoudi I. Minucci S. (2023). The simultaneous administration of microplastics and cadmium alters rat testicular activity and changes the expression of PTMA, DAAM1 and PREP. Front. Cell Dev. Biol. 11, 1145702. 10.3389/fcell.2023.1145702 36968197 PMC10033688

[B97] Vitale M. L. Akpovi C. D. Pelletier R. M. (2009). Cortactin/tyrosine-phosphorylated cortactin interaction with connexin 43 in mouse seminiferous tubules. Microsc. Res. Tech. 72 (11), 856–867. 10.1002/jemt.20771 19725064

[B116] Vogl A. W. Young J. S. Du M. (2013). New insights into roles of tubulobulbar complexes in sperm release and turnover of blood-testis barrier. Int. Rev. Cell Mol. Biol. 303, 319–355. 10.1016/B978-0-12-407697-6.00008-8 23445814

[B98] Wang C. Q. Cheng C. Y. (2007). A seamless trespass: Germ cell migration across the seminiferous epithelium during spermatogenesis. J. Cell Biol. 178 (4), 549–556. 10.1083/jcb.200704061 17698604 PMC2064462

[B99] Wang C. Q. Mruk D. D. Lee W. M. Cheng C. Y. (2007). Coxsackie and adenovirus receptor (CAR) is a product of Sertoli and germ cells in rat testes which is localized at the Sertoli–Sertoli and Sertoli–germ cell interface. Exp. Cell Res. 313 (7), 1373–1392. 10.1016/j.yexcr.2007.01.017 17359973 PMC2095131

[B100] Wei Y. Zhou Y. Long C. Wu H. Hong Y. Fu Y. (2021). Polystyrene microplastics disrupt the blood-testis barrier integrity through ROS-Mediated imbalance of mTORC1 and mTORC2. Environ. Pollut. 289, 117904. Epub 2021 Aug 3. PMID: 34371264. 10.1016/j.envpol.2021.117904 34371264

[B101] Wei Y. Zhou Y. Long C. Wu H. Hong Y. Fu Y. (2021). Polystyrene microplastics disrupt the blood-testis barrier integrity through ROS-Mediated imbalance of mTORC1 and mTORC2. Environ. Pollut. 289, 117904. 10.1016/j.envpol.2021.117904 34371264

[B102] Wen S. Zhao Y. Liu S. Yuan H. You T. Xu H. (2022). Microplastics-perturbed gut microbiota triggered the testicular disorder in male mice: Via fecal microbiota transplantation. Environ. Pollut. 309, 119789. Epub 2022 Jul 14. PMID: 35843456. 10.1016/j.envpol.2022.119789 35843456

[B103] Wong C. H. Mruk D. D. Lui W. Y. Cheng C. Y. (2004). Regulation of blood-testis barrier dynamics: An *in vivo* study. J. Cell Sci. 117 (5), 783–798. 10.1242/jcs.00900 14734653

[B104] Wong C. H. Mruk D. D. Siu M. K. Cheng C. Y. (2005). Blood-testis barrier dynamics are regulated by {alpha}2-macroglobulin via the c-Jun N-terminal protein kinase pathway. Endocrinology 146 (4), 1893–1908. 10.1210/en.2004-1464 15618353

[B105] Xiao X. Mruk D. D. Lee W. M. Cheng C. Y. (2011). c-Yes regulates cell adhesion at the blood–testis barrier and the apical ectoplasmic specialization in the seminiferous epithelium of rat testes. Int. J. Biochem. Cell Biol. 43 (4), 651–665. 10.1016/j.biocel.2011.01.008 21256972 PMC3047590

[B106] Xie X. Deng T. Duan J. Xie J. Yuan J. Chen M. (2020). Exposure to polystyrene microplastics causes reproductive toxicity through oxidative stress and activation of the p38 MAPK signaling pathway. Ecotoxicol. Environ. Saf. 190, 110133. 10.1016/j.ecoenv.2019.110133 31896473

[B107] Yan H. H. Cheng C. Y. (2005). Blood–testis barrier dynamics are regulated by an engagement/disengagement mechanism between tight and adherens junctions via peripheral adaptors. PNAS 102 (33), 11722–11727. 10.1073/pnas.0503855102 16085710 PMC1183102

[B108] Yan H. H. Mruk D. D. Lee W. M. Yan Cheng C. (2008). Blood‐testis barrier dynamics are regulated by testosterone and cytokines via their differential effects on the kinetics of protein endocytosis and recycling in Sertoli cells. FASEB J. 22 (6), 1945–1959. 10.1096/fj.06-070342 18192323 PMC2804916

[B109] Yazama F. (2008). Continual maintenance of the blood-testis barrier during spermatogenesis: The intermediate compartment theory revisited. J. Reprod. Dev. 54, 299–305. 10.1262/jrd.19169 18544902

[B111] Yin K. Wang Y. Zhao H. Wang D. Guo M. Mu M. (2021). A comparative review of microplastics and nanoplastics: Toxicity hazards on digestive, reproductive and nervous system. Sci. Total Environ. 774, 145758. 10.1016/j.scitotenv.2021.145758

[B112] Zhang T. Osborn S. Brandow C. Dwyre D. Green R. Lane S. (2013). Structured illumination-based super-resolution optical microscopy for hemato- and cyto-pathology applications. Anal. Cell Pathol. 36 (1-2), 27–35. 10.3233/ACP-130075 PMC4605645 23579249

[B113] Zhou G. X. Liu W. B. Dai L. M. Zhu H. L. Xiong Y. W. Li D. X. (2022). Environmental cadmium impairs blood-testis barrier via activating HRI-responsive mitochondrial stress in mice. Sci. total Environ. 810, 152247. 10.1016/j.scitotenv.2021.152247 34896485

[B114] Zhu Q. Li X. Ge R. S. (2020). Toxicological effects of cadmium on mammalian testis. Front. Genet. 11, 527. 10.3389/fgene.2020.00527 32528534 PMC7265816

